# 

**Published:** 1913-02-01

**Authors:** 


### Intravenous Ether Anæsthesia.

Burkhardt's ingenious system of inducing general anæsthesia by the intravenous injection of ether dissolved in normal saline solution, introduced in England with modifications by Rood, has certain manifest advantages. It has also some serious drawbacks and occasionally produces untowards results. Beresnegowsky has been investigating the action of "intravenous ether" experimentally and arrives at the following conclusions. Ether solution has an irritating effect upon the walls of blood vessels, which is a frequent cause of thrombosis in them. Pulmonary embolism is a further possibility. The solution is also detrimental to the lungs, in which it may produce swelling of the tissues, dilatation or rupture of vessels, and actual hepatisation. The kidneys and heart show changes similar to those set up by inhalation anæsthesia. This author is against intravenous ether except in certain special cases where inhalation anæsthesia is especially risky.

### QUEEN ALEXANDRA MILITARY HOSPITAL.

The Secretary of the War Office announces that the following medical men have been appointed members of the honorary consulting staff for the Queen Alexandra Military Hospital, London, for a term of four years: Mr. A. E. Barker, F.R.C.S., Sir Anthony A. Bowlby, C.M.G., F.R.C.S., Sir J. Kingston Fowler, K.C.V.O., F.R.C.P., Lieut.-Colonel P. J. Freyer, M.D. (late I.M.S.), Dr. W. S. Griffith, F.R.C.P., Dr. F. W. Mott, F.R.S., F.R.C.P., Mr. W. T. Holmes Spicer, F.R.C.S., Mr. E. B. Waggett, M.B., and Dr. A. Whitfield, F.R.C.P. The period for which the original honorary consulting staff was appointed has now expired.

### MEDICAL REFEREES FOR INDUSTRIAL DISEASES.

The Home Secretary has appointed the following as medical referees under the Workmen's Compensation Act, 1906, with a view to their being employed in cases of industrial disease (except ophthalmic cases and cases of beat hand, beat knee, and beat elbow) in various circuits where the services of a medical referee are required: Professor Sheridan Delépine and Dr. Arthur Sellers, Public Health Laboratory, York Place, Manchester; Mr. K. W. Goadby, M.R.C.S., Harley Street, W.; Mr. H. A. Schöiberg, M.B., Cardiff; and Dr. O. J. Kauffmann, Birmingham.

### Personalia.

THE REV. R. T. A. MONEY-KYRLE, rector of Ross, has been elected president of the Ross Cottage Hospital for the ensuing year, the retiring president, Mr. Guy R. Trafford, returning to the committee. Mr. J. E. S. Hewett has been re-elected vice-president.

### Appointments.

DR. F. G. CROOKSHANK, M.R.C.P. (Lond.), has been appointed physician to out-patients at the Hampstead General and North-West London Hospital.

DR. ALBERT JONES, of Ormskirk, has been recommended by the Widnes Corporation Health Committee as medical officer. He is an M.B., B.S., and D.P.H. of Liverpool University.

THE following reappointments have been confirmed at the Manchester Royal Infirmary: Fredk. H. Westmacott, F.R.C.S., assistant surgical officer to the aural department, and A. E. Woodall, M.Sc., M.D. (Vict.), medical officer at the central branch.

GENERAL HOSPITAL, BIRMINGHAM.—At a meeting of the election committee, which consists of the members of the board of management and fifty governors chosen by ballot at the annual meeting, with Mr. J. B. Clarke in the chair, Mr. R. Beatson Hird, M.D., Ch.B. (Birm.), F.R.C.S. (Edin.), M.R.C.S. (Eng.), L.R.C.P. (Lond.), was appointed honorary ophthalmic surgeon for a period of fifteen years in place of Mr. D. C. Lloyd-Owen, F.R.C.S.I., resigned.

MR. THOMAS T. RANKIN, M.B., Ch.B., M.D., at present resident medical officer at Nottingham Infirmary, has been appointed resident medical officer at Constance Road Workhouse, Camberwell. Mr. Rankin was previously senior assistant medical officer at Leeds City Hospital, assistant medical officer at Leeds Union Infirmary, and assistant medical officer at Chapel-en-le-Frith Union (indoor and outdoor). The salary and emoluments at Constance Road Workhouse amount to £355 per annum.

